# ENaC in Cholinergic Brush Cells

**DOI:** 10.3389/fcell.2018.00089

**Published:** 2018-08-15

**Authors:** Chrissy Kandel, Patricia Schmidt, Alexander Perniss, Maryam Keshavarz, Paul Scholz, Sabrina Osterloh, Mike Althaus, Wolfgang Kummer, Klaus Deckmann

**Affiliations:** ^1^Institute for Anatomy and Cell Biology, Justus-Liebig-University Giessen, Giessen, Germany; ^2^Department of Cell Physiology, Ruhr-University Bochum, Bochum, Germany; ^3^School of Natural and Environmental Sciences, Newcastle University, Newcastle upon Tyne, United Kingdom

**Keywords:** chemosensory cells, cholinergic, ENaC, urethra, urethral brush cells, salt

## Abstract

Cholinergic polymodal chemosensory cells in the mammalian urethra (urethral brush cells = UBC) functionally express the canonical bitter and umami taste transduction signaling cascade. Here, we aimed to determine whether UBC are functionally equipped for the perception of salt through ENaC (epithelial sodium channel). Cholinergic UBC were isolated from ChAT-eGFP reporter mice (ChAT = choline acetyltransferase). RT-PCR showed mRNA expression of ENaC subunits *Scnn1a, Scnn1b*, and *Scnn1g* in urethral epithelium and isolated UBC. *Scnn1a* could also be detected by next generation sequencing in 4/6 (66%) single UBC, two of them also expressed the bitter receptor Tas2R108. Strong expression of *Scnn1a* was seen in some urothelial umbrella cells and in 65% of UBC (30/46 cells) in a *Scnn1a* reporter mouse strain. Intracellular [Ca^2+^] was recorded in isolated UBC stimulated with the bitter substance denatonium benzoate (25 mM), ATP (0.5 mM) and NaCl (50 mM, on top of 145 mM Na^+^ and 153 mM Cl^−^ baseline in buffer); mannitol (150 mM) served as osmolarity control. NaCl, but not mannitol, evoked an increase in intracellular [Ca^2+^] in 70% of the tested UBC. The NaCl-induced effect was blocked by the ENaC inhibitor amiloride (IC_50_ = 0.47 μM). When responses to both NaCl and denatonium were tested, all three possible positive response patterns occurred in a balanced distribution: 42% NaCl only, 33% denatonium only, 25% to both stimuli. A similar reaction pattern was observed with ATP and NaCl as test stimuli. About 22% of the UBC reacted to all three stimuli. Thus, NaCl evokes calcium responses in several UBC, likely involving an amiloride-sensitive channel containing α-ENaC. This feature does not define a new subpopulation of UBC, but rather emphasizes their polymodal character. The actual function of α-ENaC in cholinergic UBC—salt perception, homeostatic ion transport, mechanoreception—remains to be determined.

## Introduction

Bitter, sweet, umami, salty, sour, and fatty are the six recognized taste qualities detected by taste buds (Chaudhari and Roper, 2010). In type II sensory cells in the oropharyngeal taste buds, bitter, sweet, and umami perception is mediated by the canonical taste transduction signaling cascade, including G protein-coupled taste receptors, the taste-specific G protein α-gustducin, phospholipase Cβ2 (PLCβ2), and the transient potential receptor cation channel subfamily M member 5 (TRPM5) (Chaudhari and Roper, 2010). Other classes of G protein-coupled receptors respond to short- and long-chain fatty acids (Chaudhari and Roper, 2010). In contrast, acid (protons) and salt (sodium chloride) are monitored by ion channels, directly leading to depolarization of the taste cell. Nonselective cation channels formed by polycystic kidney disease 2-like 1 protein (PKD2L1) and polycystic kidney disease 2-like 3 protein (PKD1L3) were proposed as candidates for sour taste receptors (Huang et al., 2006; Ishimaru et al., 2006; Lopezjimenez et al., 2006; Chaudhari and Roper, 2010). An ion channel that is long been thought to mediate salt perception is the amiloride-sensitive epithelial sodium channel, ENaC (Heck et al., 1984; Avenet and Lindemann, 1988; Lindemann et al., 1998; Lin et al., 1999; Lindemann, 2001; Chandrashekar et al., 2010). It is predominantly expressed in epithelial cells of the colon, lung, kidney, sweat and salivary glands, where it is a major regulator of sodium absorption and, thereby, essential for fluid homeostasis (Duc et al., 1994; McDonald et al., 1995; Garty and Palmer, 1997). ENaC is also expressed in the urothelium (Carattino et al., 2005; Du et al., 2007; Birder et al., 2010; Birder and Andersson, 2013). The canonical heteromeric ion channel consists of three subunits (α, β, γ) (Canessa et al., 1994b), encoded by the genes *Scnn1a, Scnn1b*, and *Scnn1c*. A fourth δ-subunit with distinct characteristics was identified and the presence of this subunit changes the biophysical characteristics as well as molecular regulation of this ion channel. Mice, however, lack a functional gene for this subunit and its physiological function remains unclear (Giraldez et al., 2012; Wichmann et al., 2018). ENaC is a constitutively active ion channel. Still, its expression, membrane abundance and open probability are tightly regulated by extrinsic and intrinsic factors. These include hormones, intracellular kinases and intramembrane lipids, as well as the extracellular sodium concentration, pH and mechanical stimuli (Chraïbi and Horisberger, 2002; Althaus et al., 2007; Baines, 2013; Kleyman et al., 2018). The ion conductivity of αβγ-ENaC is limited to monovalent cations (Li^+^ > Na^+^ > K^+^) (Kellenberger and Schild, 2002).

Extraoral chemosensory cells, monitoring the composition of the mucosal lining fluid, have been described in the respiratory, gastrointestinal and urogenital tract. Like type II taste cells, they express the canonical taste transduction signaling cascade (taste receptors, α-gustducin, PLCβ2, TRPM5) (Höfer et al., 1996; Höfer and Drenckhahn, 1998; Finger et al., 2003; Krasteva et al., 2011, 2012; Deckmann et al., 2014; Schütz et al., 2015). They respond to bitter substances and bacterial products with a release of acetylcholine and initiate avoidance reflexes, thereby apparently serving as sentinels situated at entrances into the body (Finger and Kinnamon, 2011; Lee and Cohen, 2015; Deckmann and Kummer, 2016). These cholinergic epithelial cells also express villin, a structural protein of microvilli. Such cells have originally been termed “brush cells” in the respiratory tract, and this term has also been adopted to the villin-positive, cholinergic chemosensory cells of the urethra (urethral brush cells = UBC) (Deckmann et al., 2014, 2015). In line with the sentinel concept, UBC respond to heat-inactivated uropathogenic *Escherichia coli* and are connected to sensory nerve fibers (Deckmann et al., 2014). Bitter application into the urethral lumen reflexively triggers enhanced detrusor activity, which has been interpreted as a protective reflex, as potential hazardous content is expelled from the urethra through micturition (Deckmann et al., 2014; Kummer and Deckmann, 2017).

Most cholinergic UBC are polymodal chemosensory cells, responding both to bitter substances and to glutamate with an increase in intracellular calcium concentration ([Ca^2+^]_i_) (Deckmann et al., 2014). This discriminates them from type II taste bud cells, which are generally responsive either to bitter, representing an aversive stimulus, or to umami, an attractive stimulus (Nelson et al., 2001; Chaudhari and Roper, 2010). At the urethral mucosa, both stimuli represent a potential danger signal, since many bacterial products have bitter quality and glutamate (umami) facilitates bacterial growth in urine. Here, we aimed to determine whether their polymodal properties extend beyond taste receptor mediated qualities, focusing upon the perception of salt.

## Materials and methods

### Animals

Mice expressing enhanced green fluorescent protein (eGFP) under the control of the promoter of the acetylcholine synthesizing enzyme, choline acetyltransferase, (ChAT-eGFP; B6.Cg-Tg(RP23-268L19-EGFP)2Mik/J; Stock No. 007902) were obtained from Jackson Laboratory (Bar Habor, ME, USA). Mice expressing tdTomato, a bright red fluorescent protein, under the control of the promotor of *Scnn1a*, the coding gene sequence of α-ENaC (*Scnn1a*/tdTomato; Guy et al., 2015) were kindly provided by J. Guy and J. Staiger (Institute for Neuroanatomy, University Medical Center Goettingen, Georg-August-University Goettingen, Germany). This study was carried out in accordance with the recommendations of European Communities Council Directive of 24th November 1986 (86/609/EEC). The protocol was approved by the local authorities (Animal Welfare Officer at the University of Giessen and the Committee for Animal Welfare, Dept. V54, Regierungspräsidium Giessen, Germany; reference no. 572_M).

### Cell isolation

Cell isolation was performed as described previously (Deckmann et al., 2014). In brief: Urethrae were dissected, cut into small pieces, and enzymatically digested in dispase (2 mg/mL; Sigma-Aldrich/Merck, Darmstadt, Germany) and trypsin/PBS (1:1, Invitrogen, Carlsbad, CA, USA). After mechanical dissociation, cells were separated through a cell strainer (pore size 70 μm; BD Bioscience, Franklin Lakes, NJ, USA). The ChAT promotor is constitutively active in cholinergic chemosensory cells (Tallini et al., 2006). Hence, UBC constitutively express eGFP which served to sort them via FACS and to identify them with a fluorescence microscope.

### RT-PCR

Total RNA from dissected urethra or pooled isolated cells (*n* = 4 samples, sorting based on ChAT-eGFP expression by FACS; BD FASCARIA III cell sorter, settings and analysis were performed with a BD FACSDiva v6.1.3; BD Bioscience, Franklin Lakes, NJ, USA) was extracted using the Qiagen RNeasy Micro Kit (Qiagen, Hilden, Germany) according to the manufacturer's protocol. Extracted total RNA from kidney was used as positive control. RT-PCR was performed as described previously (primer sequences: Table S1; Deckmann et al., 2014).

### Next generation sequencing

Next generation sequencing was performed as described elsewhere (Scholz et al., 2016). In brief: isolated single eGFP-positive cells were identified, picked and transferred to a PCR tube using a combined confocal laser-scanning/patch-clamp setup (Leica TCS SP5, Leica Microsystems/Luigs-Neumann, Wetzlar/Ratingen, Germany). Cell lysis, cDNA generation and amplification were performed using the Sigma SeqPlex RNA Amplification Kit (Sigma-Aldrich/Merck, Darmstadt, Germany). For library preparation, the Illumina Nextera XT DNA sample preparation protocol (Part # 15031942 Rev. C) was used. Samples run together with a 2 × 75 bp read length using the MiSeq Reagent Kit v3 (150 cycles) and the Illumina MiSeq Desktop Sequencer (Illumina, San Diego, CA, USA). The sequencing reads were aligned to the mm9 reference genome and transcriptome using TopHat2 (2.0.9). The TopHat output files were saved in BAM format and evaluated by Cuffdiff2 (2.1.1). All samples were compared and evaluated in one calculation cycle, allowing the algorithm to estimate the Fragments Per Kilobase Million (FPKM) values at the transcript level resolution and to control for variability across the replicate libraries.

### Immunohistochemistry and whole-mount immunostaining

Specimen preparations and analyses were performed as described previously (Krasteva et al., 2011). In brief: urethrae used for immunohistochemistry (*N* = 3) and gall bladders used for whole-mount immunostaining (*N* = 2) were fixed using transcardiac perfusion with Zamboni solution (2% paraformaldehyde/15% saturated picric acid in 0.1 M phosphate buffer, pH 7.4). Fixed organs were dissected, washed in 0.1 M phosphate buffer (0.1 M NaH_2_PO_4;_ 0.1 M Na_2_HPO_4_), and either incubated overnight in 18% sucrose in 0.1 M phosphate buffer and frozen in liquid nitrogen or mounted on a block of silicon elastomer using insect pins. Primary antibody was applied to 4–18 tissue sections from every individual animal. Primary antibodies were chicken anti-RFP (NBP1-97371, 1:200 dilution; Novus Biologicals, Littleton, CO, USA) and rabbit anti-TPRM5 (1:2,000) (Kaske et al., 2007). Secondary antibodies were goat-anti rabbit Ig conjugated to Alexa 488 (1:500; Thermo Fisher Scientific Inc. Waltham, MA, USA) and donkey-anti chicken Ig conjugated to Cy3 (1:2,000; Dianova, Hamburg, Germany). Nuclei were labeled with 4′,6-diamidino-2-phenylindol (DAPI; 1 μg/ml; Sigma-Aldrich/Merck, Darmstadt, Germany). All sections were rinsed and coverslipped with carbonate-buffered glycerol (pH 8.6). Sections were evaluated by epifluorescence microscopy (Axioplan 2, Zeiss, Wetzlar, Germany) or with a confocal laser scanning microscope (LSM 710, Zeiss, Wetzlar, Germany). Specificity of secondary reagents was validated by omission of primary antibodies.

### Measurement of intracellular calcium concentration

Measurement of intracellular calcium concentration ([Ca^2+^]_i_) was performed as described previously (Deckmann et al., 2014). In brief: Isolated cells were loaded with the fluorescent calcium indicator Calcium Orange® AM (0.01 μg/μL; Thermo Fisher Scientific Inc., Waltham, MA, USA) and plated on coverslips. [Ca^2+^]_i_ was analyzed with a confocal laser scanning microscope (LSM 710 with ZEN 2010 B SP1, Zeiss, Wetzlar, Germany). Lasers and filters were: eGFP: excitation with Argon laser at 488 nm; recording of emission at 495–553 nm with optical filters MBS-488/561/633; Calcium Orange: excitation with DPSS561-10 laser at 561 nm; recording of emission at 566-683 nm with optical filters MBS-558/561. Regions of interest were selected manually and fluorescence intensities at the start of the recording period were set arbitrarily at 100%. Test stimuli and concentrations were denatonium benzoate (25 mM; Molekula, Munich, Germany), ATP (0.5 mM; Sigma-Aldrich/Merck, Darmstadt, Germany) and NaCl (1–150 mM; Carl Roth, Karlsruhe, Germany), and inhibitors and controls included the osmolarity control mannitol (1–150 mM; Sigma-Aldrich/Merck, Darmstadt, Germany) and the ENaC inhibitor amiloride (0.01–100 μM; Sigma-Aldrich/Merck, Darmstadt, Germany). All recordings were done during continuous superfusion with Tyrode III buffer (NaCl 130 mM; HEPES 10 mM; glucose 10 mM; KCl 5 mM; MgCl_2_ 1 mM; CaCl_2_ 8 mM; sodium pyruvate 10 mM; NaHCO_3_ 5 mM; 2.5 mL/min; 37°C). Stimuli were added under continuous flow of Tyrode III into the chamber, so that indicated concentrations were reached initially and then washed out. Since baseline concentration of Na^+^ in the buffer was 145 mM, the total concentration after addition of 1–150 mM ranged from 146 to 295 mM.

### Statistical analysis

Data were analyzed for normal distribution by the Kolmogorov-Smirnov test. Multiple comparison analysis was performed by Kruskal-Wallis test followed by Dunn's Multiple Comparison Test. *P* ≤ 0.05 were regarded as statistically significant. Analyses were performed by GraphPad Prism 5 (GraphPad Software Inc., La Jolla, CA, USA).

## Results and discussion

**Figure 1:**
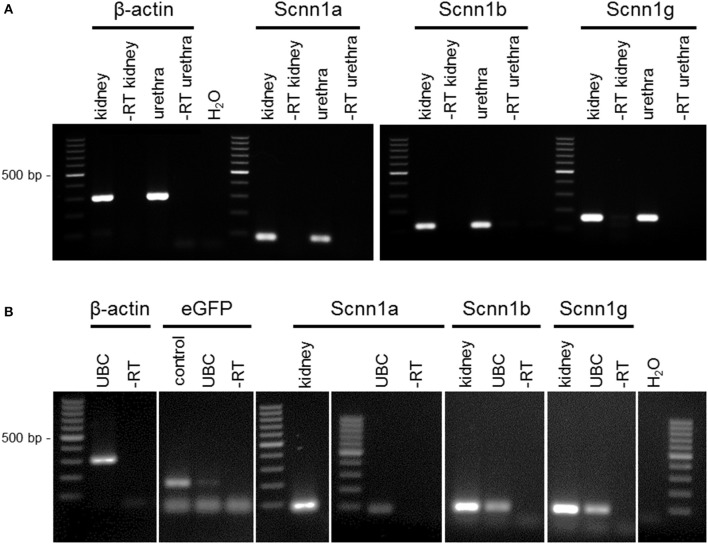
RT-PCR of urethral epithelium and isolated UBC. **(A)** RT-PCR of urethral epithelium. **(B)** RT-PCR of UBC. Cells were isolated and sorted based on ChAT-eGFP expression by flow cytometry; agarose gel; α-ENaC (*Scnn1a*; 82 bp), β-ENaC (*Scnn1b*; 115 bp), γ-ENaC (*Scnn1g*; 150 bp), β-actin (300 bp), eGFP (180 bp); ± RT = aliquots processed with/without reverse transcription; kidney = positive control; H_2_O = water control.

**Figure 2:**
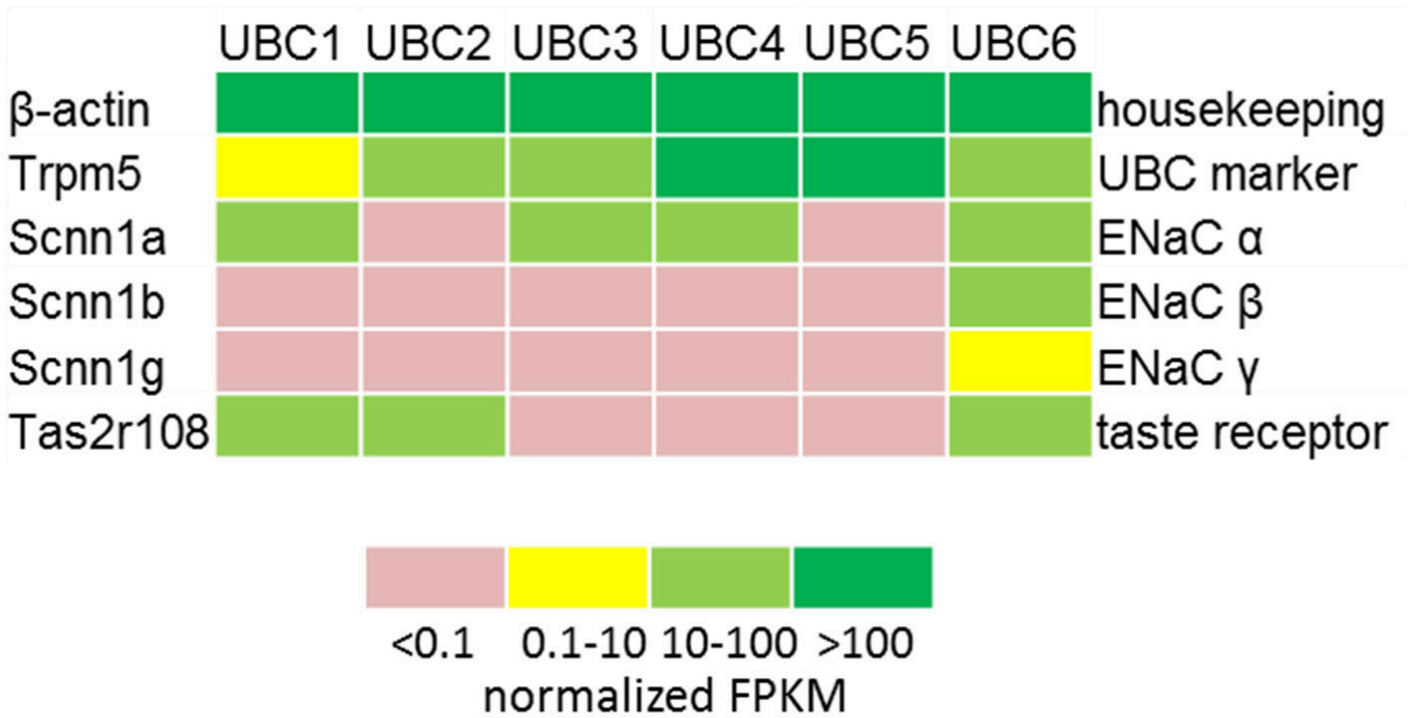
Expression of ENAC genes and markers of chemosensory cells in single UBC determined by NGS. Heatmap displaying the detection levels as normalized FPKM in GFP-positive UBC. FPKM, Fragments Per Kilobase Million.

RT-PCR revealed mRNA expression of the ENaC subunits *Scnn1a, Scnn1b*, and *Scnn1g* in the urethral epithelium (Figure 1A) and in pooled isolated UBC (Figure 1B). Next generation sequencing (NGS) of six isolated single eGFP-positive cells showed a heterogeneous expression pattern of *Scnn1a, b, g* (Figure 2). *Scnn1a* was detected in 4/6 cells (66.6%), *Scnn1b* and *Scnn1g* only in 1/6 cells. Canonical ENaC is composed of the α-, β-, and γ-subunit (Canessa et al., 1994a), but the ENaC α-subunit alone is able to form amiloride-sensitive homomers *in vitro* (Canessa et al., 1994b).

To further validate *Scnn1a* expression in cholinergic UBC, urethral tissue sections of a *Scnn1a* reporter mouse strain were labeled for cholinergic UBC. In view of often experienced methodological problems in detecting ChAT by immunohistochemistry in peripheral cells, we set out to establish a technically more reliable marker for immunohistochemical detection of cholinergic UBC. Villin-antibodies, an often used marker for brush cells in general, appeared not suitable for this purpose as there is a considerable number of villin-positive but ChAT- and TRPM5-negative slender epithelial cells in the murine urethra, in addition to the villin/ChAT/TRPM5-positive cells (Deckmann et al., 2014). These two phenotypes represent truly different cell populations, since genetic ablation of the transcription factor Skn-1a/Pou2f3 selectively prevents the development of TRPM5-positive (i.e., cholinergic UBC) but not of villin-positive but TRPM5-negative urethral cells (Yamashita et al., 2017).

**Figure 3:**
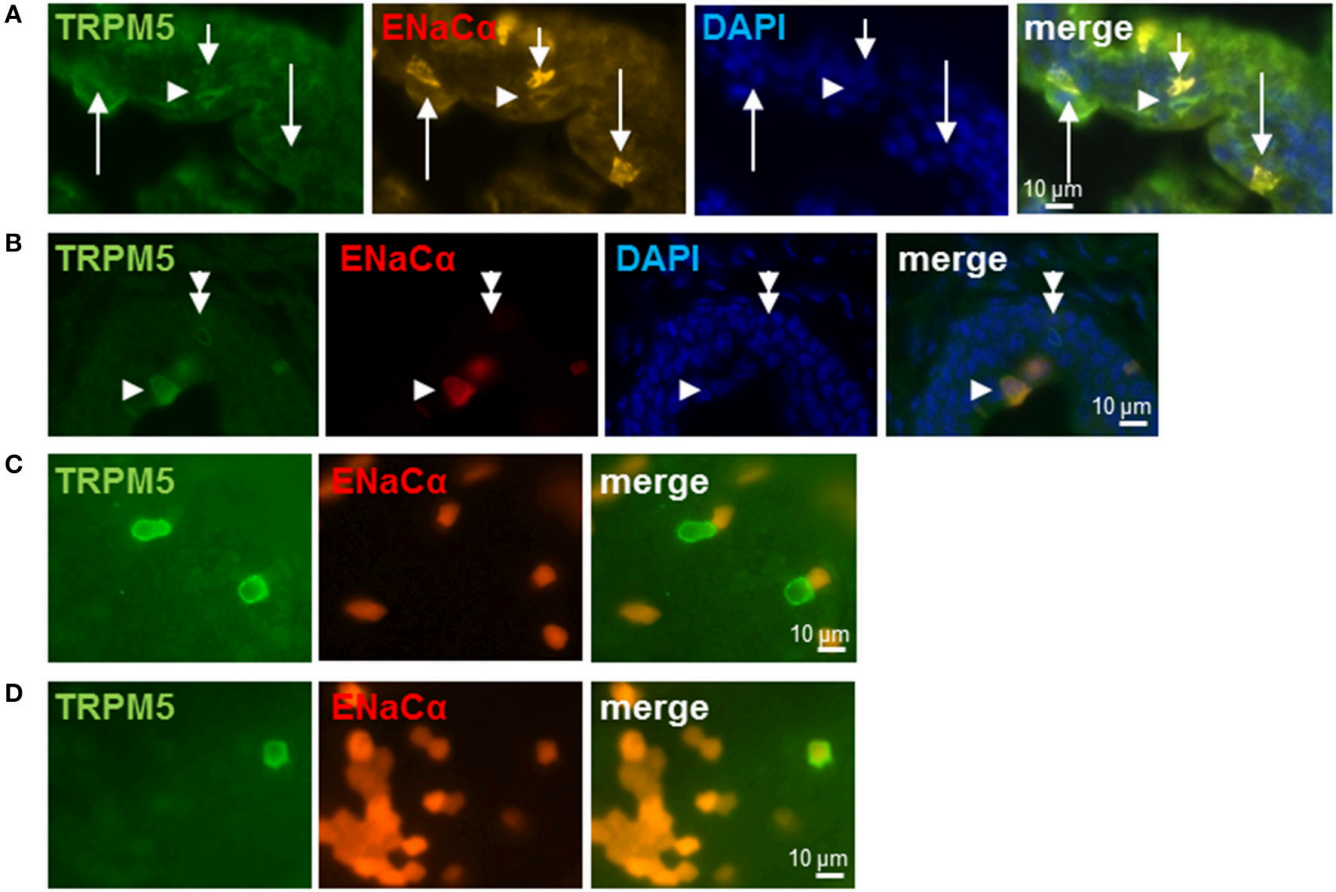
Brush cells express ENaCα. **(A,B)** Urethra; frozen sections: *Scnn1a*/tdTomato (orange or red), TRPM5-immunoreactivity (green) and cell nuclei (DAPI; blue). Several epithelial cells express α-ENaC only (*arrows* in **A**). At least two of them (*long arrows* in **A**) are urothelial umbrella cells as judged by their shape and position. *Arrowheads* point to α-ENaC (orange) and TRPM5 (green) double-positive cells, the *doubled arrowhead* points to a TRPM5-immunoreactive cell that does not express α-ENaC. **(C,D)** Gall bladder; whole-mount: *Scnn1a*/tdTomato (red) and TRPM5 (green). **(C)** Selected cells show no co-localization of α-ENaC and TRPM5. **(D)** Selected TRPM5-immunoreactive cell shows co-localization of α-ENaC and TRPM5.

We used TRPM5-immunolabeling as a marker for cholinergic UBC in *Scnn1a*-tdTomato reporter mice. In these mice, strong expression of *Scnn1a* was observed in several cells of the urethral epithelium (Figures 3A,B). Among them were umbrella cells, which build up the luminal lining in the proximal parts of the urethra being covered with an urothelium and which can be readily identified by virtue of their position and morphology. This is in line with the previously reported ENaCα-immunoreactivity at the luminal membrane of umbrella cells in the rat urinary bladder (Smith et al., 1998) and functional investigation of this cell type (McCloskey et al., 2017). Notably, this cell layer did not consistently express tdTomato with positive and negative umbrella cells occurring in a mosaic pattern (Figure 3A). Although heterogeneity of umbrella cells with respect to other characteristics such as uroplakin expression has also been reported in select localizations such as the human ureter (Riedel et al., 2005), this labeling pattern might reflect incomplete expression of tdTomato in potentially *Scnn1a*-expressing cells. To test for this possibility, we looked for tdTomato expression in the gall bladder whose mucosal surface is known for homogeneous ENaCα expression (Li et al., 2016). In two gall bladder whole-mount preparations, strong tdTomato expression was observed in epithelial cells covering only about 21% (case 1: 26.8%, case 2: 16.0%) of the mucosal surface whereas nearly 80% remained unlabeled (Figures 3C,D). Gall bladder whole-mounts were also incubated with TRPM5-antibody in order to label cholinergic chemosensory brush cells that are also present in this epithelium (Schütz et al., 2015). Two out of 69 TRPM5-positive cells expressed *Scnn1a*-tdTomato (Figures 3C,D).

Among non-umbrella cells with nuclei located in deeper layers of the urethral epithelium, we detected co-localization of TRPM5-immunoreactivity and *Scnn1a*-tdTomato signal (30 of 46 TRPM5-positive cells, 65%, *N* = 3 animals) (Figures 3A,B) as well as TRPM5-positive cells without *Scnn1a*-tdTomato signal (Figure 3B). These observations support our findings in RT-PCR experiments and single cell sequencing that a subpopulation of UBC expresses α-ENaC.

**Figure 4:**
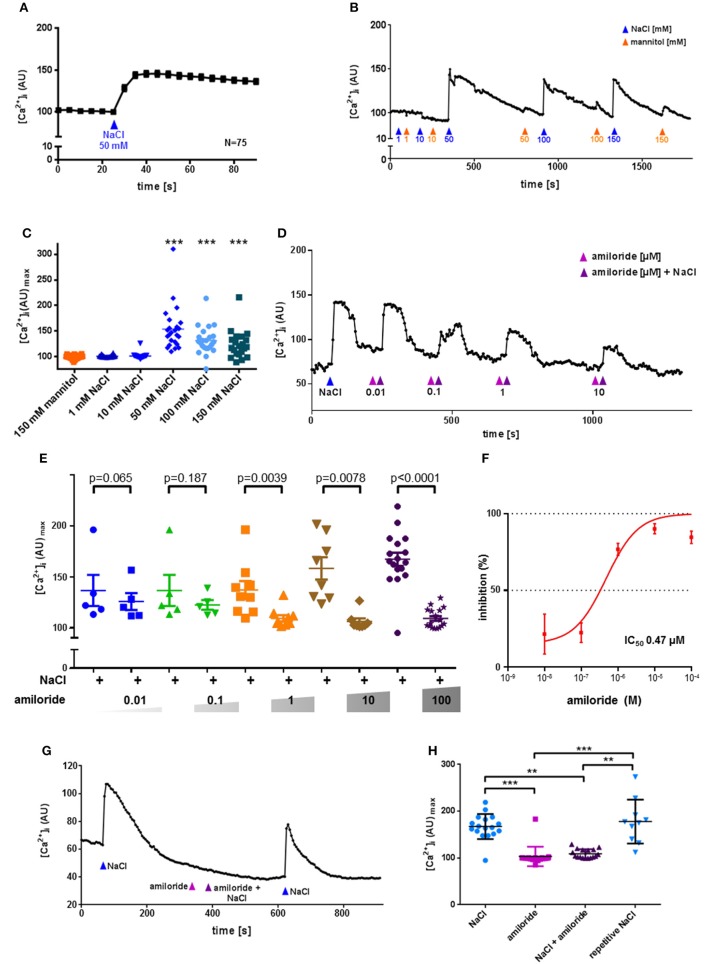
UBC response to NaCl is ENaC dependent and not an osmolarity effect. Urethral epithelial cells of ChAT-eGFP reporter mice were isolated and UBC were identified due to eGFP fluorescence. Experiments were performed during continuous superfusion with Tyrode III buffer containing 145 mM Na^+^. NaCl, mannitol and amiloride were added under continuous flow of Tyrode III into the chamber, so that indicated concentrations were reached initially and then washed out. In case of NaCl, concentration changes on top of the baseline of concentrations in Tyrode III are indicated. Thus, the total sodium concentration after addition of 1–150 mM ranged from 146 to 295 mM. Y-Axis depicts arbitrary units (AU) of Calcium Orange® fluorescence recorded by confocal laser scanning microscopy, correlating to [Ca^2+^]_i_. **(A)** NaCl evokes an increase in [Ca^2+^]_i_; shown are mean and SEM. **(B)** Representative recording of changes in Calcium Orange® fluorescence in a single cholinergic (eGFP^+^) UBC in response to increasing concentrations of NaCl (blue arrowhead) and mannitol (orange arrowhead). **(C)** Depicted are peak values after stimulation with mannitol (150 mM, *N* = 22) and NaCl (1 mM, *N* = 22; 10 mM, *N* = 22, 50 mM, *N* = 24, 100 mM, *N* = 23; 150 mM, *N* = 21); ^*^P < 0.05, ^**^P < 0.01, ^***^P < 0.001 compared to mannitol 150 mM, Kruskall-Wallis test followed by Dunn's Multiple Comparison Test. **(D)** Representative recording of changes in Calcium Orange® fluorescence in a single cholinergic (eGFP^+^) UBC in response to NaCl (50 mM) in absence and presence of increasing concentrations of amiloride (0.01–10 μM). **(E)** Depicted are peak values after application of NaCl (50 mM) in absence and presence of increasing concentrations of amiloride (0.01 μM, *N* = 5; 0.1 μM, *N* = 5; 1 μM, *N* = 9; 10 μM, *N* = 8; 100 μM, *N* = 17), *p*-values were calculated by, Kruskal-Wallis test and are indicated in the figure. **(F)** Dose–inhibition curve of amiloride in UBC. IC_50_ = 0.47 μM. **(G)** Representative recording of changes in Calcium Orange® fluorescence in a single cholinergic (eGFP^+^) UBC in response to NaCl (50 mM) in absence and presence of amiloride (0.1 mM). **(H)** Depicted are peak values after application of NaCl (50 mM, *N* = 17), amiloride (0.1 mM, *N* = 17), NaCl (50 mM) together with amiloride (0.1 mM, *N* = 17), and repetitive NaCl (50 mM, *N* = 10); ^*^P < 0.05, ^**^P < 0.01, ^***^P < 0.001, Kruskal-Wallis test followed by Dunn's Multiple Comparison Test.

Functionally, application of NaCl evoked significant increases in [Ca^2+^]_i_ in 70% of the isolated cholinergic UBC. At concentrations of 50, 100, and 150 mM NaCl, but not at 1 or 10 mM, significant increases in [Ca^2+^]_i_ were observed (Figures 4A–C). There was a tendency toward a decline in the evoked increase in [Ca^2+^]_i_ with increasing NaCl concentration. To test for a possible osmolarity effect, increasing concentrations of mannitol were administered alternating with corresponding concentrations of NaCl (Figure 4B). Since mannitol had no stimulatory effect upon [Ca^2+^]_i_ even at 150 mM (Figure 4B), the observed reaction of UBC to 50 mM NaCl, which was used for further characterization of polymodality, is not an osmolarity effect. Amiloride, an ENaC inhibitor that also suppresses salt perception in type I taste cells of the taste buds (Vandenbeuch et al., 2008), fully blocked the [Ca^2+^]_i_ response in UBC to NaCl (50 mM) with an IC_50_ of 0.47 μM (Figures 4D–F). Even at the highest concentration used (100 μM), the inhibitory effect of amiloride was reversible upon wash-out (Figures 4G,H). Thus, the NaCl-induced increase in [Ca^2+^]_i_ in UBC is amiloride-sensitive. This, however, does not seem to involve the canonical αβγ-ENaC which was shown to detect low concentrations of sodium in taste receptor cells of fungiform papillae in mice (Chandrashekar et al., 2010). First, only 1/6 UBC expressed all three ENaC subunits detected by NGS. Second, activity of mouse αβγ-ENaC appears to be maximal at 60 mM extracellular sodium (Sheng et al., 2004), a concentration which is below the stimuli used in the present study (≥50 mM NaCl added to Tyrode III). Third, this response to the NaCl stimulus was sensitive to amiloride, whereas baseline [Ca^2+^]_i_ in UBC (in the presence of 145 mM Na^+^ in Tyrode III) was not (Figure 4G). Fourth, even though this study used calcium-imaging and this does not directly measure ENaC-activity, the IC_50_ for the observed inhibition of the calcium signal by amiloride is above that reported for inhibition of mouse αβγ-ENaC (0.1 μM) (Ahn et al., 1999). This might suggest an alternative amiloride-sensitive cation channel containing the ENaC α-subunit. Recently, it was shown that α-ENaC can assemble with alternative ion channels such as the acid sensing ion channel 1 (Jeggle et al., 2015), which may form a non-selective cation channel (Trac et al., 2017). The expression of acid sensing ion channels in UBC was, however, low and inconsistent (Figure S1). Alternatively, α-ENaC can form homomeric ion channels *in vitro* (Canessa et al., 1994b). Their physiological function, however, remains to be proven *in vivo*.

Irrespective of the molecular composition of the amiloride-sensitive sodium conductance in UBC, exposure to high concentrations of NaCl might trigger a membrane depolarization which may stimulate calcium-influx via voltage-gated calcium channels and subsequent release of acetylcholine. UBC showed consistent expression of α-subunits of the L-type voltage-gated calcium channels Ca_v_1.2 (*Cacna1d*), Ca_v_1.3 (*Cacna1d*), and Cav2.3 (*Cacnac1e*) (Figure S2). Furthermore, there was strong expression of the auxiliary subunit β4 (*Cacnb4*) in 5/6 cells. The auxiliary β-subunits are generally important for membrane expression and the β4-subunit seems to determine subcellular membrane-localization in polarized cells (Campiglio and Flucher, 2015). Voltage-gated calcium channels thus represent promising targets for the coupling of NaCl-induced membrane depolarization to acetylcholine release in UBC.

The physiological meaning of salt responsiveness of cholinergic UBC remains uncertain. In adult C57BL/6J mice, urinary sodium concentration is around 150 mM, similar to our 145 mM baseline in Tyrode buffer, and can significantly increase during water deprivation or high-salt intake (Li et al., 2012). During such conditions, cholinergic UBC may thus be exposed to sodium concentrations which trigger calcium responses as shown in this study. UBC are interpreted as sentinels of the lower urinary tract equipped for monitoring the mucosal surface for potential hazardous content, especially bacterial products (Deckmann et al., 2014; Deckmann and Kummer, 2016; Kummer and Deckmann, 2017). Threatening bacterial infections, however, are usually not connected to increased salt concentrations. Thus, α-ENaC may here serve other functions than monitoring luminal NaCl concentration. Canonical ENaC holds a key position in maintaining electrolyte and water homeostasis, e.g., concentration of primary urine in the kidney (Kellenberger and Schild, 2002). Given the low number of cholinergic UBC in the urethra and their minimal exposure to the luminal surface, this function appears rather unlikely for this particular cell type. However, ENaC is also a mechanosensitive ion channel, reacting to shear stress (Althaus et al., 2007; Guo et al., 2016). This opens the possibility that ENaC-subunit carrying UBC may be involved in sensing urine flow in the urethra. Notably, as mechanical strain affects the entire epithelium and is not restricted to the luminal membrane, it will reach UBC without a clear connection to the luminal surface (“closed type,” see Figure 3B and Deckmann and Kummer, 2016). Cholinergic UBC are connected to sensory nerve fibers and, reflexively, initiate micturition in response to a bitter stimulus in the urethral lumen (Deckmann et al., 2014). This has been interpreted as a protective reflex in that potentially hazardous content will be flushed out (Deckmann et al., 2014; Kummer and Deckmann, 2017). Voiding efficiency is augmented by sensory feedback from the urethra, where flow sensors are physiologically well characterized but not yet defined anatomically (Todd, 1964; Peng et al., 2008; Danziger and Grill, 2015). Thus, mechanosensitivity of cholinergic UBC may serve to augment the reflex response they have initiated.

**Figure 5:**
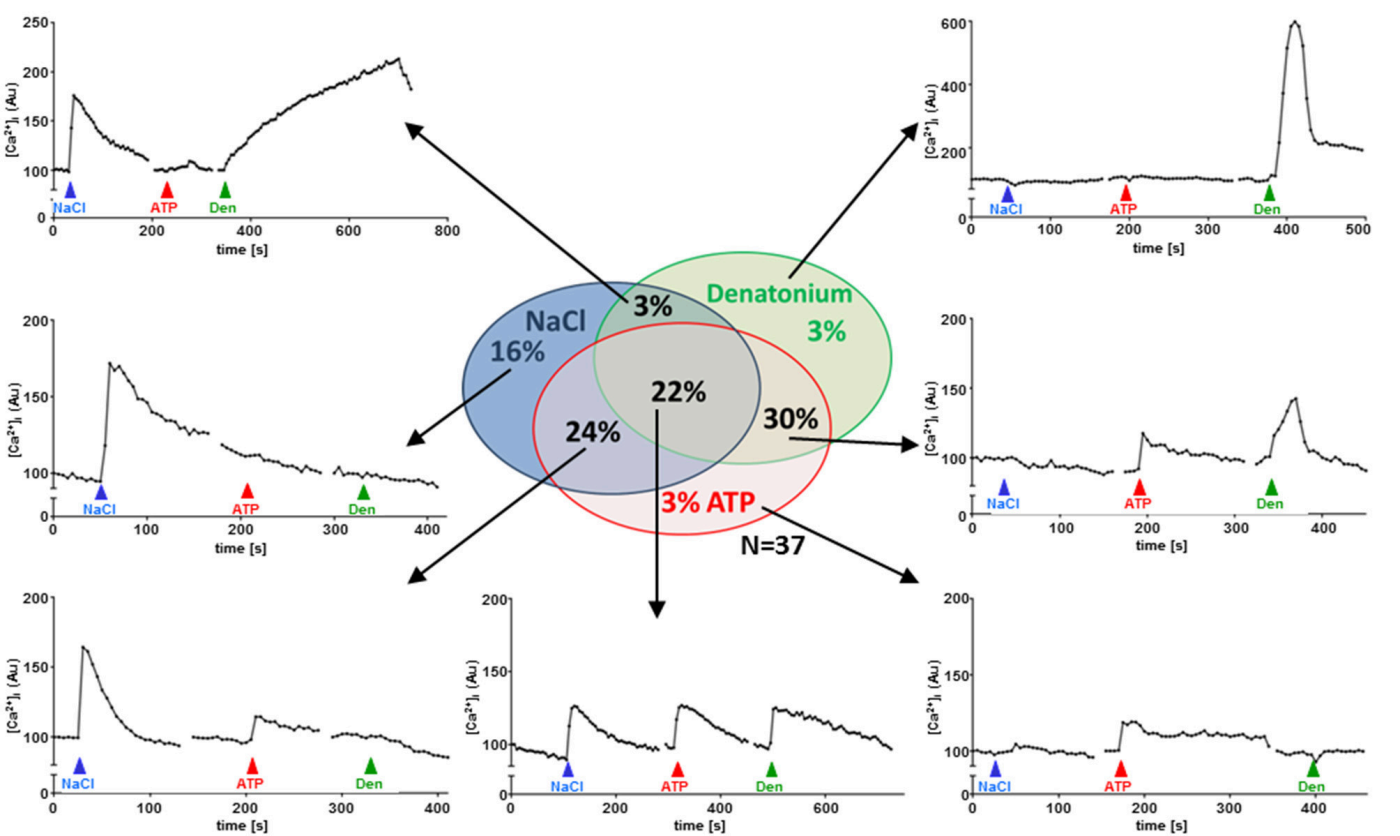
UBC responses to NaCl, ATP and denatonium. Sequential stimulation with NaCl (50 mM on top of baseline concentrations in Tyrode), ATP (0.5 mM) and denatonium (25 mM); sequence of stimuli was changed between experiments (*N* = 37). Graphs show representative recordings of changes in Calcium Orange® fluorescence in single cholinergic (eGFP^+^) UBC. Experiments were performed during continuous superfusion with Tyrode III buffer Stimuli were added under continuous flow of Tyrode III into the chamber, so that indicated concentrations were reached initially and then washed out. Y-Axis depicts arbitrary units (AU) of Calcium Orange® fluorescence.

To test for polymodal properties, cholinergic UBC were successively exposed to NaCl and ATP (*N* = 90; 70% responded to NaCl), to NaCl and denatonium (*N* = 36; 67% responded to NaCl), and to all three stimuli (*N* = 37; 65% responded to NaCl, Figure 5). When responses to both NaCl and denatonium were tested on 36 UBC, all three possible response patterns occurred in a balanced distribution (Figure 5): 42% NaCl only, 33% denatonium only, 25% both stimuli. These percentages are roughly reflected by the (immuno)histochemical (65% of UBC expressing *Scnn1a*-tdTomato signal) and by the NGS data with 4/6 cells (67%) expressing *Scnn1a*, and 2 of them (33%) expressing additionally a known receptor for denatonium, i.e., Tas2r108 (Figure 2). Of course, the small total number of cholinergic UBC subjected to NGS (*N* = 6) precludes a systematic quantitative analysis.

We have previously shown that a substantial number of denatonium-responsive UBC also reacts to monosodium glutamate (Deckmann et al., 2014). In terms of oropharyngeal gustation, these substances reflect an aversive (denatonium: bitter) and an attractive (monosodium glutamate: umami) stimulus, and, accordingly, are perceived by distinct cell populations, which still are considered as subtypes of type II taste cells (Chaudhari and Roper, 2010). The present data show an even broader diversity of UBC properties in that some of them share features also with type I cells of taste buds, expressing ENaC and being responsive to NaCl (Vandenbeuch et al., 2008). These findings further substantiate the polymodal character of cholinergic UBC. As far as further distinctive criteria are missing, we interpret the multiple combinations of responsiveness to various chemosensory stimuli and gene expression of related signaling components as phenotypic variation of a broadly tuned, polymodal chemosensory cell rather than defining multiple, clearly separated cell types.

## Conclusion

In sum, we could show that a fraction of cholinergic UBC expresses α-ENaC and responds to the salty stimulus NaCl in an amiloride-sensitive manner. This feature does not define a new subpopulation of UBC, but rather emphasizes their polymodal character.

## Author contributions

KD designed research and performed statistical analysis. KD, CK, PatS, PauS, MK, and SO performed research and analyzed data. KD and WK obtained funding. KD, WK, AP, and MA drafted the manuscript. Work was supervised by WK and KD.

### Conflict of interest statement

The authors declare that the research was conducted in the absence of any commercial or financial relationships that could be construed as a potential conflict of interest.

## Abbreviations

AU: arbitrary units
ChAT: choline acetyltransferase
Den: denatonium benzoate
eGFP: enhanced green fluorescent protein
ENaC: epithelial sodium channel
[Ca^2+^]_I_: intracellular calcium concentration
TRPM5: transient receptor potential cation channel subfamily M member 5
UBC: urethral brush cell.
