# Supplementary material for: ENaC in Cholinergic Brush Cells
Source: Front Cell Dev Biol. 2018 Aug 15;6:89. doi: 10.3389/fcell.2018.00089 (PMC6103785; doi:10.3389/fcell.2018.00089)
Supplement: Supplementary file 1 [file Table_S1.DOCX]

**Table S1**

| Gene | Primer | Product length, bp | GenBank accession no. |
| --- | --- | --- | --- |
| Scnn1a | For GCAGTTTGCTTTGCTGTTCGAGGA  Rev AGACCAGCTTGTCCGAATTGAGGT | 82 | NM_011324.2 |
| Scnn1b | For TCCAGGCCTGTCTTCATTCCTGTT  Rev TGGGAAGTCCCTGTTGTTGCAGTA | 115 | NM_001272023.1 |
| Scnn1g | For TGACCTGCTTCTTCGATGGGATGT  Rev ACTTGCAGACCATACTCACTGCCT | 150 | NM_011326.3 |
| β-2-Microglobulin | For ATTCACCCCCCACTGAGACTG  Rev GCTATTTCTTTCTGCGTGCAT | 192 | NM_009735 |
| β-actin | For GTGGGAATGGGTCAGAAGG  Rev GGCATACAGGGACAGCACA | 300 | NM_007393.2 |
| eGFP | For AAG TTC ATC TGC ACC ACC G  Rev TCC TTG AAG AAG ATG GTG CG | 180 |  |

**Table S1 - Primers used for RT-PCR.** *Scnn1*: Official symbols for gene names sodium channel, nonvoltage-gated 1 a-b-g (ENaCαβγ); bp = base pairs; For = forward, Rev = reverse
