# Supplementary figures and images for: ENaC in Cholinergic Brush Cells
Source: Front Cell Dev Biol. 2018 Aug 15;6:89. doi: 10.3389/fcell.2018.00089 (PMC6103785; doi:10.3389/fcell.2018.00089)

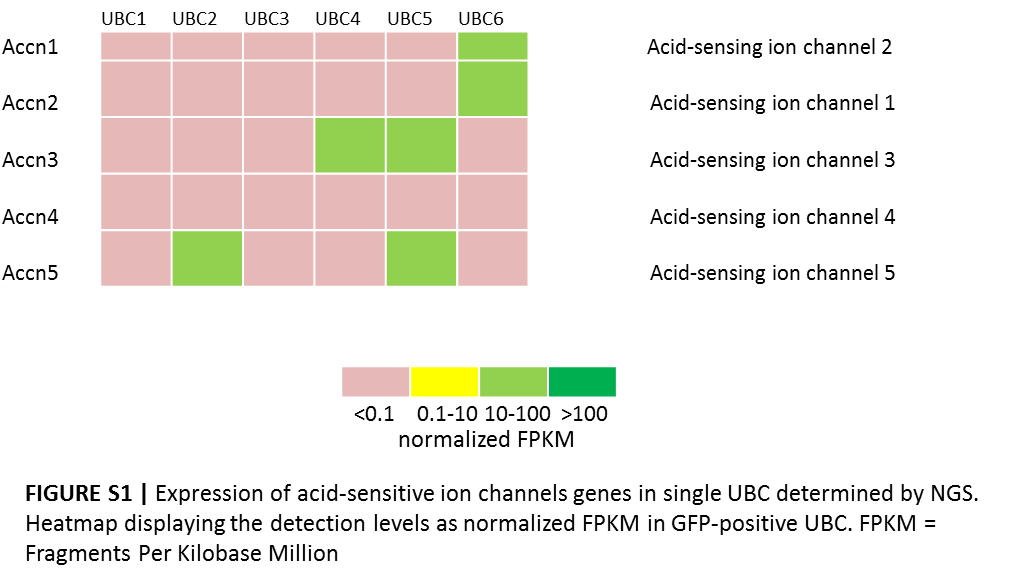

Supplement: Supplementary file 2 [file Figure_S1.TIF]

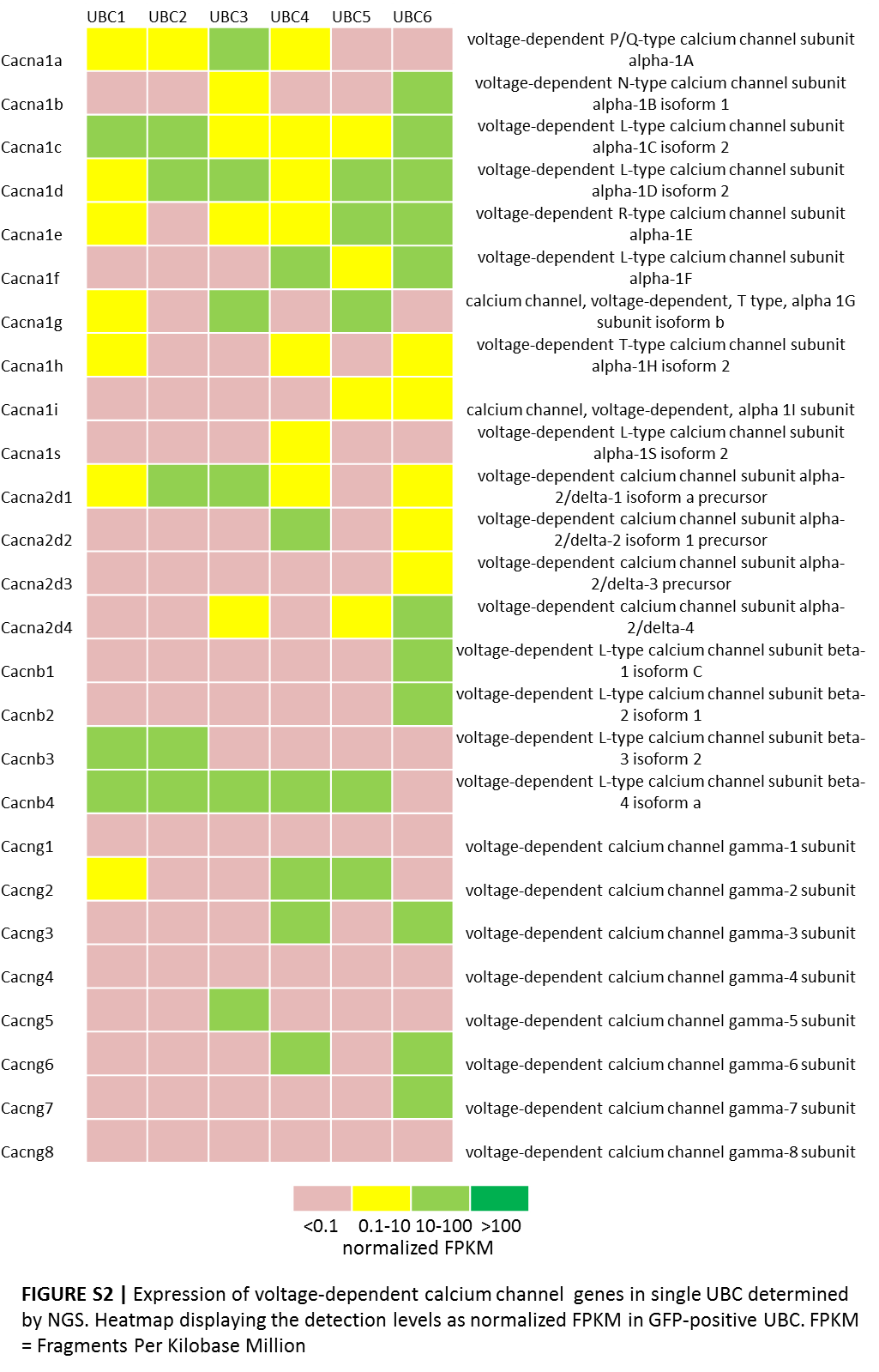

Supplement: Supplementary file 3 [file Figure_S2.TIF]
